# Supplementary material for: A Brazilian Rare-Disease Center’s Experience with Glucosylsphingosine (lyso-Gb1) in Patients with Gaucher Disease: Exploring a Novel Correlation with IgG Levels in Plasma and a Biomarker Measurement in CSF
Source: Int J Mol Sci. 2024 Mar 1;25(5):2870. doi: 10.3390/ijms25052870 (PMC10931658; doi:10.3390/ijms25052870)
Supplement: Supplementary file 1 [file ijms-25-02870-s001.zip › ijms-2861593-supplementary.pdf]

(SUPPLEMENTARY MATERIAL)

2.1. Case 1–Treatment naïve Gaucher Disease type 1

Patient 1 is a 47-year-old female diagnosed with GD type 1 at the age of 46 years. She was born to a consanguineous couple of Ashkenazi Jewish ancestry, and has 3 healthy siblings. The patient has a positive family history of Parkinsonism. She was referred to the GD Reference Center at the Hospital de Clínicas de Porto Alegre (HCPA), Brazil, after a long diagnostic odyssey that included the diagnosis of many autoimmune disorders such as arthralgia, Raynaud syndrome, Ankylosing spondylitis, and Lupus. The patient also presented with hyperferritinemia (ferritin = 1,059 ng/mL) not controlled with the use of immunosuppressors, and anemia that worsened during her pregnancy. Exome sequencing reported a homozygous pathogenic variant in the *GBA1* gene c.1226A>G; p.Asn409Ser (N370S) confirming the diagnosis of GD1. Laboratory exams at admission showed hemoglobin levels of 11.9 g/dL, leukocyte count at 7,340 cells/mm3, platelets at 161,000/mm3, Cht activity of 2,076 nmol/h/mL (NRV = 8.8–132), and lyso-Gb1 of 395 nmoL/L. Abdominal ultrasonography revealed, apart from a 1.1 cm hemangioma in the hepatic right lobe, normal liver and spleen volumes. GCase activity was 0.92 nmol/h/mg prot in leukocytes (NR = 10–45 nmol/h/mg prot). At diagnosis, the Disease Severity Scoring System (DS3) was 3.25/19 (mild = < 3.00; moderate = 3.00–5.99; marked = 6.00–19) and the Severity Score Index (SSI) was 4/49 (mild = 0–10; moderate = 11–19; severe ≥20). The patient started treatment with imiglucerase 15UI/kg biweekly at the age of 47 years. Four months after the start of the treatment, it was noted a decrease of the lyso-GB1 value to 172 nmoL/L as shown in figure 3A. This patient continues her follow-up in our center, with a current SSI score of 4 (Zimran Severity Score Index, categorized as mild = 0–10; moderate = 11–19; severe ≥20) and a DS3 score of 3.25 (Disease Severity Score, classified as mild = < 3.00; moderate = 3.00–5.99; marked = 6.00–19).

2.2. Case 2: Treatment naïve Gaucher Disease type 2

Patient 2 is a 7-month-old male patient diagnosed with GD2 at the age of 5 months. He was born at 38 weeks gestational age from uncomplicated pregnancy with birth weight 3,315g (74th centile CDC growth charts). In the first days of life, the patient was diagnosed as having congenital CMV. It was then noticed hepatosplenomegaly, elevation of liver transaminases and thrombocytopenia. No seizures were reported by the family or reports from the primary care. At the age of 3 months, the patient was transferred to our hospital. Laboratory tests at admission showed hemoglobin at 10 g/dL, ferritin 3,403 ng/mL (NRV = 23 a 336 ng/mL), platelets at 53,000/mm3, Cht activity of 12,235 nmol/h/mL (NRV = 8.8–132), and lyso-Gb1 of 542 nmoL/L. Abdominal ultrasound showed an enlarged liver and spleen (4.6 cm in length) with normal echotextures. The molecular diagnosis confirmed that the patient was homozygous for the pathogenic variant c.1448T>C; p.Leu483Pro (L444P). GCase activity of 0.86 nmol/h/mg protein in leukocytes (NR = 10-45 nmol/h/mg protein) As the patient exhibited evidence of disease burden ERT was recommended and initiated at 5 months of age with Imiglucerase (Cerezyme®) 60UI/kg biweekly. His weight at 5 months was 6.6kg (Z score -0.9). Two months after the start of the treatment it was noticed an increase of the lyso-GB1 value to 1,212 nmoL/L as shown in figure 3B. There was no change in the patient's weight from the beginning of the treatment. The patient passed away at the age of 8 months due to sepsis of unclear etiology and suspicion of Hemophagocytic Lymphohistiocytosis Syndrome (HLH).

2.3. Case 3: Treatment naïve Gaucher Disease type 3

Patient 3 is a two-year-old male born to a non-consanguineous couple after an uncomplicated pregnancy, with a full-term birth weight of 2996 g. The family history is noncontributory. The patient's motor development was considered appropriate for his age, and no seizures were reported in the first months of life. An initial work-up with an oncologist at the age of 4 months revealed hepatosplenomegaly, and bone marrow findings suggestive of GD. Laboratory tests at admission to our center showed hemoglobin at 6.7 g/dL, leukocyte count at 5,070 cells/mm^3, platelets at 96,000/mm^3, ferritin of 156 ng/mL, Cht activity of

12,136 nmol/h/mL (NR = 8.8–132), and lyso-Gb1 of 3,447 nmoL/L. Physical evaluation revealed marked hepatosplenomegaly and abnormal horizontal saccadic eye movements with bilateral convergent strabismus. Abdominal ultrasound showed an enlarged liver and spleen (15.5 cm in length) with normal echotextures and atelectasis of the pulmonary's inferior lobes. The molecular diagnosis confirmed that the patient was homozygous for the pathogenic variant c.1448T>C; p.Leu483Pro (L444P). GCase activity was 1.3 nmol/h/mg protein in leukocytes (NR = 10-45 nmol/h/mg protein). The patient initiated treatment with imiglucerase at 60 IU/kg biweekly at the age of 2 years old. Lyso-Gb1 levels decreased to 1,474 nmol/L after 11 months of treatment, as shown in Figure 3C. Interestingly, there was a discrepancy in this case between the Cht and lyso-Gb1 levels after the beginning of treatment, showing a decrease in lyso-Gb1 values (from 3,447 to 1,593 nmol/L) and an increase in Cht levels (from 12,136 to 21,538 nmol/L). The only contributing factor at the time of these measurements was a severe sinus infection that the individual experienced. This patient continues his follow-up in our center, with a current SSI score of 26 (Zimran Severity Score Index, categorized as mild = 0–10; moderate = 11–19; severe ≥20) with improvement of his splenomegaly on physical examination.

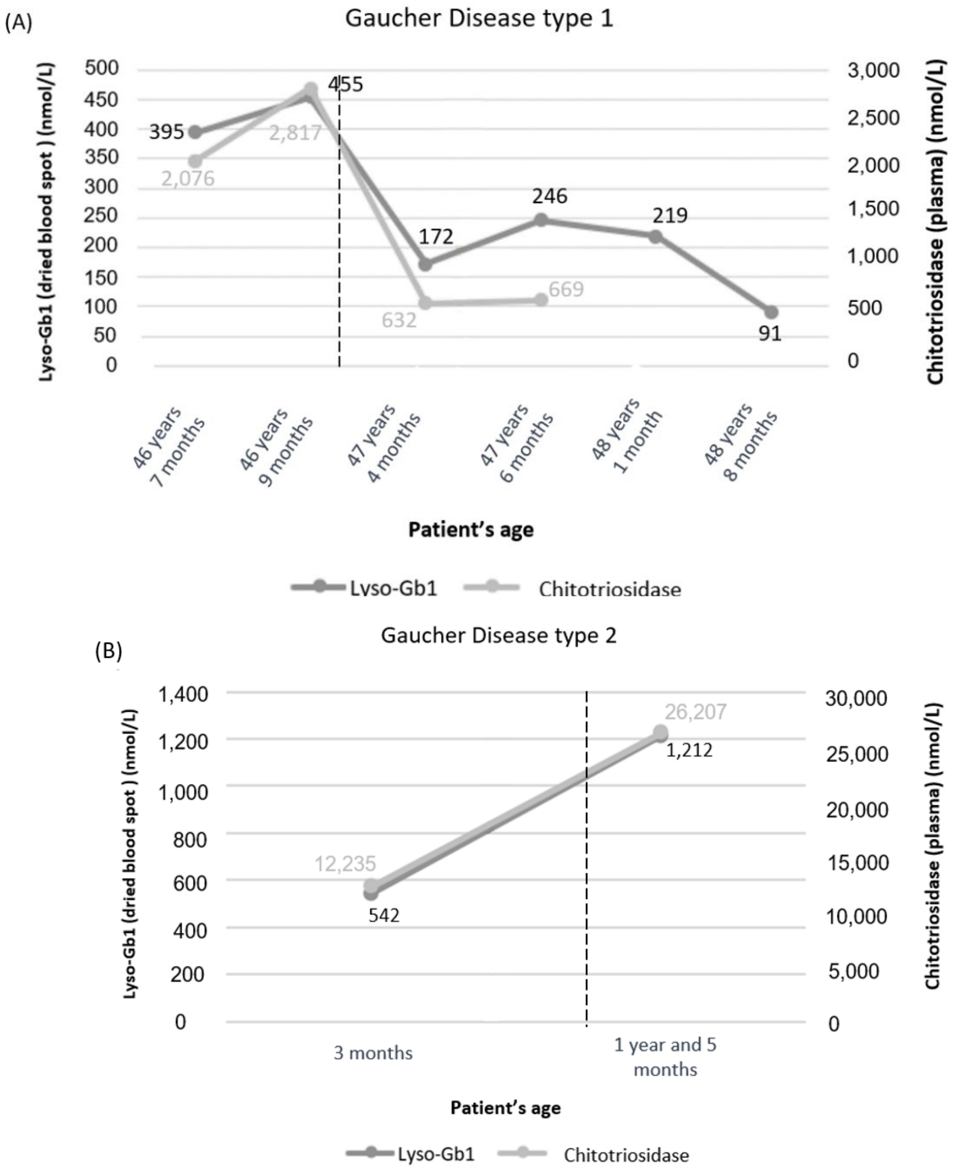

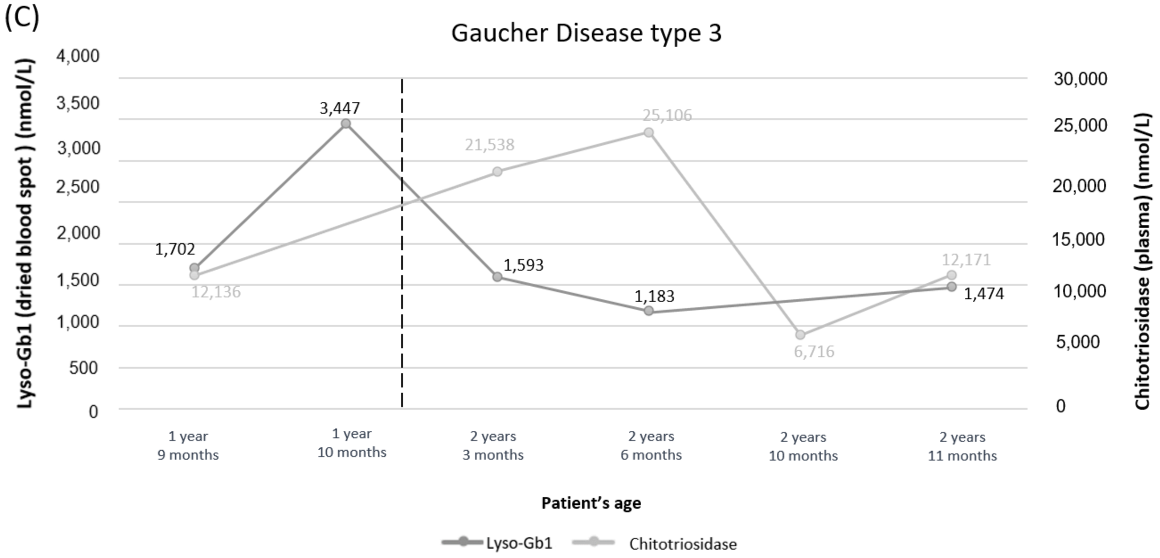

Figure S1. Biomarker values in (A) Patient 1, (B) Patient 2 and (C) Patient 3. Vertical dotted line denotes start of treatment with ERT.
